# Dating furniture and coopered vessels without waney edge – Reconstructing historical wood-working in Austria with the help of dendrochronology

**DOI:** 10.1016/j.dendro.2013.11.002

**Published:** 2014

**Authors:** Andrea Klein, Sebastian Nemestothy, Julia Kadnar, Michael Grabner

**Affiliations:** University of Natural Resources and Life Sciences Vienna, Austria

**Keywords:** Dendrochronology, Historical wood-working, Furniture, Coopered vessels

## Abstract

In the present study, 208 furniture and 168 coopered vessels from three Austrian museums were examined. Dendrochronology was used to date objects and to extract further information such as the necessary time for seasoning, wood loss through wood-working and methods of construction. In most cases sampling was done by sanding the cross section and making digital photographs using a picture frame and measuring digitally.

The dendrochronological dates of the sampled furniture range between 1524 and 1937. The group of furniture includes cupboards, chests, tables, benches, commodes and beds. In many cases furniture was artfully painted and sometimes even shows a painted year. With the help of dendrochronology it was proved that some objects had been painted for some time after construction, or had been over-painted. Most furniture, however, was painted immediately after completion. In this case, the seasoning and storage time of the boards and the wood loss due to shaping can be verified. As an average value, 14 years have passed between the dendrochronological date of the outermost ring and the painting. The time span includes time of seasoning and storage and the rings lost by wood-working. This leads, on the one hand to a short storage time of less than 10 years and on the other hand to very little wood loss due to manufacturing. Those boards being less shaped turned out to be back panels of cupboards, therefore they are recommended to be sampled for dating.

Coopered vessels were dated between 1612 and 1940. There was evidence that staves were split and not sawn in many cases. The staves were often split out of the outermost part of the tree and hardly any wood was worked away which was proved by the close dendrochronological dates of the single staves of a vessel.

Since there is a short time of storage and only little wood loss through wood-working, dating of objects without a waney edge becomes reasonable.

## Introduction

Wood-working includes many different crafts, each having its own rules and traditions. In the present study the Austrian traditions of the furniture and coopered vessel production are illuminated with the help of dendrochronology.

Wood-working craftsmen have always been specialised to the one or other craft. However, in Austria it was not until the late 14th century, that all these specialists were combined in the professional craft (“Zunft”) of carpentry. By 1382 joinery and cooperage became separate crafts and started to develop and to specialise their way of working (Radkau and Schäfer, 1987).

### Country furniture

In Austria, a line can be drawn between furniture produced for urban and courtly use, and for rural use. In this investigation only country furniture is included. It was generally produced by three different circles: professional joiners, mobile wageworkers and farmers themselves (Moser, 1949). In some cases, endowment-furniture was bought on the local market, but most furniture was made by wageworkers or farmers (Moser, 1947). The group of furniture produced domestically by farmers is often hard to distinguish. Through the centuries, farmers have developed their own skills and traditions. Often, folkloristic dating can be difficult, because there is still a lot of uncertainty (Moser, 1949). The design did not follow any style trend, but their own tradition, that often did not change for a long time (Moser, 1949). It has been asserted that joinery methods hardly changed over centuries in the Tyrol (Colleselli, 1968). Furthermore, from the 13th and 14th century on, farmers started to gain sophisticated skills and knowledge about recognisable wood-working techniques (Moser, 1949). This makes the distinction between furniture produced by farmers and by joiners difficult today.

Furniture has often been embellished with some kind of painting and in Austria the famous, colourful, baroque surface-painting of wooden furniture started at the end of the 17th (Lipp, 1964). Today, paintings can help to date furniture from a folkloristic point of view, but sometimes this does not date the production of the furniture itself. Furniture was part a wedding endowment. Often it was specially produced, but sometimes old furniture was over painted for this occasion (Bader, 1998). Lipp (1986) mentions professional painters not being wood-workers at all, but working on old unpainted cupboards.

### Coopered vessels

To save transportation costs, the roughing out of staves took place in the woodland, where stems were debarked, split (Voigt, 1930) and air-dried (Grünn, 1968). The moister content which can be reached by natural drying is about 15% (Bosshard, 1975). To reach this moisture content, the coopers calculated half a year per one centimetre for softwood and one year per one centimetre for hardwood (Grünn, 1968; Kindler, 1949). A vast amount of high quality wood was needed for the coopered vessel production (Radkau and Schäfer, 1987). Until the middle of the 20th century, most coopered vessels were produced by splitting (Kindler, 1949).

### Possibilities of dendrochronology

Dating objects like furniture and vessels often does not lead to an absolute date of the construction year. First, some years or tree rings get lost by wood-working and second, boards were seasoned for some years before use (Waldner, 2005).

Nevertheless, dendrochronology leads to interesting discussions. On the one hand the age of the furniture can be proved; on the other hand historical wood-working processes might be reconstructed. Eckstein (2007) states that the work of the dendrochronologist is the translation of information stored in tree rings into human language. This describes quite well, the aim of the present study. There is some “translation work” already done for panel-paintings (e.g. Eckstein et al., 1986) and musical instruments (e.g. Beuting, 2011), however only few analyses have been made on country furniture (Waldner, 2005, Thun and Alsvik, 2009) or historic coopered vessels (Waldner, 2006) up to now.

### Hypotheses

At the beginning of the work four hypotheses were stated:

1. For many centuries, harvesting timber was under strict regulations in Austria (Johann, 1994). The large amount of wood which was needed for mining activities led to strongly varying regional differences in wood availability (Johann, 1968). Consequently wood was a valuable commodity in historical times and as little wood as possible was removed by the wood-working process. Therefore the dendrochronogical dates without waney edge are close to the construction date. This can be verified by searching for boards showing a waney edge, by focusing on the shape of the board and by referring to the region where the board was cut out of the tree.
2. Drying times were much shorter than expected. The seasoning and storage was never longer than one generation, usually even less. This is the second precondition for the high expressiveness of the dendrochronogical dates and can be proved by dating objects with a painted year.
3. Folkloristic dating does not always meet the construction period. In some cases furniture was over painted, leading to wrong year of manufacturing.
4. Boards or staves of one object were often sawn or split out of a single tree. This hypothesis can be verified by internal dating. It will give a hint on working methods. One assumption was that one tree was chosen to split out all staves for the production of a vessel. Waldner (2006) proved this previously when dating a mediaeval vessel.

## Material and methods

Furniture and vessels from three Austrian museums have been analysed. One museum is located in Malta in Carinthia (Bauernmöbelmuseum, Probstkeusche), in the very southern part of Austria, another one is located in Stainz in Styria (Landwirtschaftsmuseum), in the south-eastern part of Austria, and most objects belonging to the Austrian Open Air Museum in Stübing, close to Graz in Styria, where houses and objects from all over Austria were available.

Sampled furniture includes cupboards, chests, tables, beds and commodes. The wood-working method and the thickness of the boards varies greatly between the furniture from different regions. Most of the cupboards and chests have been painted in the local tradition. Folkloristic dating was not part of the analysis. However, if the painted furniture was dated, it was helpful to interpret dendrochronological results.

The term “vessel” includes casks and open vats of different size, most being kept together with a wooden ring. Open vats have a wall thickness of 10–20 mm, casks have a thickness of 30–40 mm.

All museum objects were prepared for measurement in situ and never left their location. First, the cross-section was sanded. Unfortunately, sanding itself cannot be seen as non-destructive. Hence a lot of effort was set on selecting the boards. Sanding furniture was either done on non-painted or non-visible boards. Vessels were sanded on the bottom part of the staves.

Direct measurement of the rings on the radial surface would have been non-destructive, but the innermost rings were often distorted if the log had not been sawn directly through the pith, and some surface preparation was often required.

**Fig. 1:**
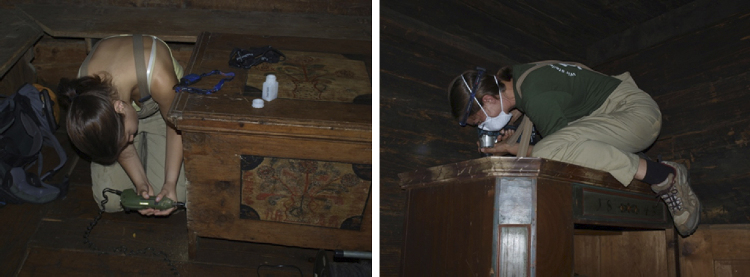
Sanding country furniture with a precision bore grinder.

This preparation was carried out using a small precision bore grinder, having a spindle collar of 20 mm in diameter was used. The sanding paper of the same size was glued to the collar in grain size 120, 240 and 600. Two pictures of sampling are shown in Fig. 1.

**Fig. 2:**
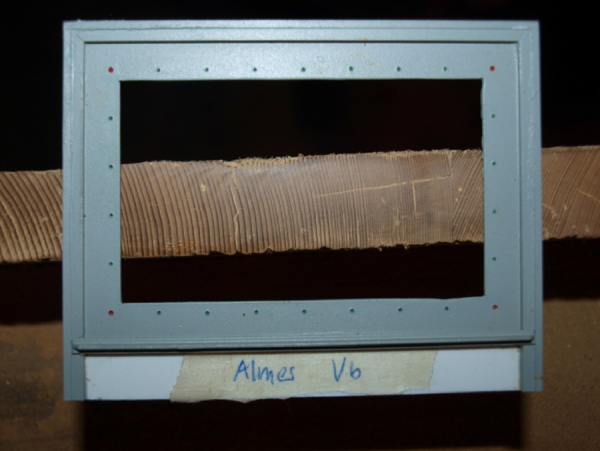
The reference frame applied on a sideboard of a chest.

After sanding, the sample was photographed using a scaled frame to make calibration possible before tree ring measurements. Furthermore the reference frame guarantees a constant distance between the object and the lens, which is necessary, if the board was too big for the frame and more pictures had to be overlapped digitally. The picture-frame was self-made of plastic and brass profiles with scaling dots applied in 10 mm distance, as shown in Fig. 2. The inner size of the frame was chosen at 75 mm × 45 mm, the frame bridge with the scaling dots was produced in 12 mm width. This size of the frame seemed to be most suitable – not too big, so that very narrow rings could be measured in high resolution and not too small, so that only a few pictures of one board had to be taken. Only if the cross-section was not accessible and the board was precisely radially orientated, the radial surface was photographed, using the picture frame as well.

The pictures were made on an Olympus E-420 digital camera, using a 25 mm focal length lens causing the smallest possible distortion. The distance of approximately 120–150 mm between the object and the camera was determined by the lens and the size of the reference frame.

If pictures had to be overlapped to get one picture of the whole board, this was done using the program Adobe Photoshop CS5. Before that, distortion caused by the lens had to be corrected. This was done by using four red dots, which are positioned in exact rectangular shape and an accurately defined distance to each other on the reference frame. Horizontal and vertical offset were also corrected by the same way using the lens correction feature of Adobe Photoshop CS5. The ring width was then measured on the picture, using the measuring program WinDENDRO™ 2009 (Regent Instruments Canada Inc.). Calibration was done by defining a horizontal and vertical scale of the known distance given by the reference frame. Tree ring series of all vessels and furniture were dated using *t*-value and Gleichläufigkeit generated by TSAP (www.rinntech.com) and by visual checking of the plots. First, all boards belonging to the same object were cross dated against each other. Then the average curve of the object and the single curves were cross dated against the Austrian chronologies (Geihofer et al., 2005).

As many different boards or staves as possible were sanded of one object. By limitation of less possible destruction, often only two or three boards could be sampled, but in other cases almost a full set was measured.

**Fig. 3:**
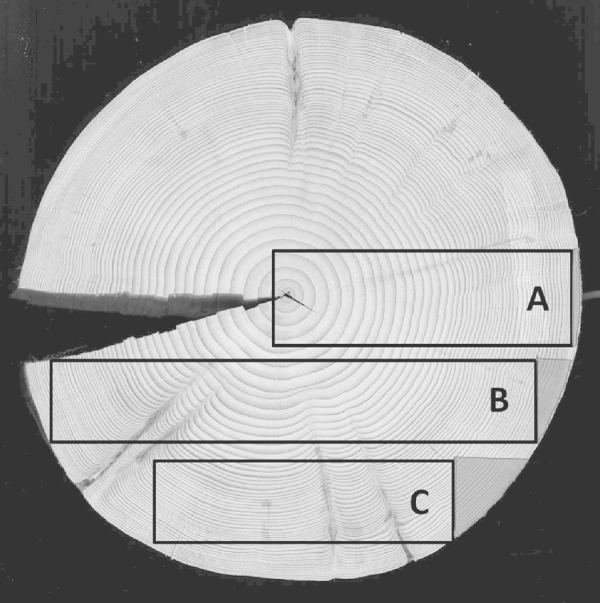
Three different board types, as they were cut out of the trunk. Type A shows the lowest wood loss (coloured in grey).

To prove the hypotheses that only a little wood was lost by working, the orientation of the board or stave in the tree trunk was verified by looking on the photographs. Three different positions were distinguished, as shown in Fig. 3.

If furniture with a painted year was dated, the number of lost tree-rings as well as the corresponding distance of lost wood (in mm) was examined. Therefore the mean ring width of the sample was multiplied by the number of years missing to the year painted. If a waney edge had been present on these painted examples, the exact seasoning time could have been calculated. Unfortunately, this was never the case.

To verify the threshold from which *t*-value on boards or staves might be sawn or split from the same trunk a literature search was done first. Beuting (2011) mentions that the following criteria have to be met to argue that two pieces of wood come from a single tree: *t*-values of at least 8, Gleichläufigkeit of at least 70 and at least 70 years of overlap. To get the threshold of *t*-values for Austrian trees, internal cross dating was done. Three sites of living trees in Austria (at least 15 trees per site) of each species were analysed. All trees in one site were sampled twice and internally cross dated, so that characteristic *t*-values for two samples originating from the same tree could be given.

## Results

In total, 442 staves from 59 casks and 109 open vats, as well as 505 boards from 80 cupboards, 89 chests, 12 tables, 15 benches, eight commodes and four beds were analysed.

### Wood species

The wood species identified in the analysed objects are shown in Table 1. Country furniture was made of softwood, with more than 50% being made out of Norway spruce. Some 25% of the vessels were made of oak, whilst the remaining 75% were made from various softwood species.

### Dating

In total, 52% of all sampled furniture and 42% of all sampled vessels were successfully dated. To be more precise, 283 boards from 107 pieces of furniture were dated between 1524 and 1937. The oldest furniture is a chest from Carinthia and the youngest is a table from Vorarlberg showing a waney edge. Furthermore 211 staves from 70 vessels were dated between 1612 and 1940.

### Seasoning time

**Fig. 4:**
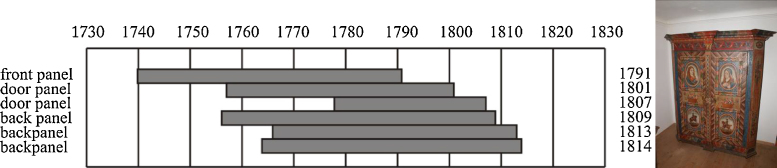
Dendrochronologically dated cupboard with the painted year 1819.

Focusing on furniture having a painted year, 20 objects could be dated. Table 2 lists all dendrochronological dates of the youngest furniture board and the painted year. Six of them were painted some time after construction, or were over-painted some time later, with the range of dates covering from 78 to almost 300 years later. 14 boards were painted only a short time after the furniture was constructed. The time between the outermost tree ring and the initial painting was between two years and 31 years. On average 14 years or 19.5 mm were left from the final ring of the dendrochronological date to the painted year. Unfortunately, it is not possible to distinguish whether this time difference is a result of seasoning, or wood loss in shaping the wood (see below). It is possible to give some hints – like in Fig. 4, where the difference between the dendrochronological dates is only five years (see below).

### Wood loss due to wood-working

In 17 pieces of furniture all different types of boards, as back boards, side boards, front boards and door frames, have been sampled. The amount of wood lost in board production was investigated. Within the 17 objects, 13 back panels, one front panel, one door panel and one chest lid turned out to be the elements with the least wood loss. Furthermore, the backboards often exhibited a trapezoidal shape (such as the grown tree stem), which none of the other board types did. This indicates that only little wood was cut away, especially in the case of backboards.

Fig. 4 gives an example of a painted cupboard. The painted year was 1819, the youngest date was the date of a back panel having its last tree ring dated in 1814. Only five years or 6.6 mm wood were lost between the last ring and the painted date. Door panels are usually more heavily processed and therefore they show an earlier dendrochronological date. The front panel was cut with a bevel so that again lot of wood was lost by wood-working.

Focusing on coopered vessels, the dendrochronological dates of the final tree rings of different staves within a single vessel, range within a few years. A clear statement about having little wood loss due to working could be verified either by counting sapwood rings or by having staves showing a waney edge. Unfortunately, most of the staves were made of spruce which does not form any colour heartwood and none of the staves showed a waney edge. The fact, however, that dendrochronological dates range only within a few years indicates that only very little wood was removed.

**Fig. 5:**
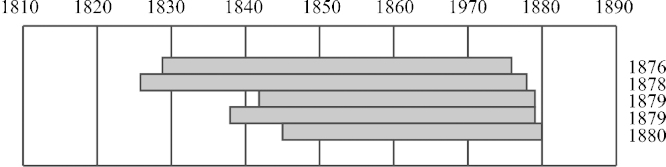
Dendrochronological dates of five staves of one vessel.

**Fig. 6:**
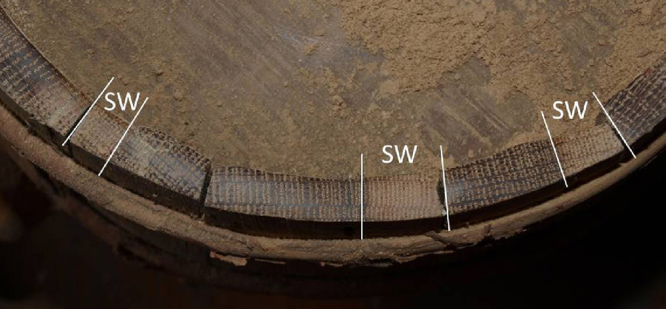
Picture of the dated vessel of Fig. 5. The sapwood formation is marked with the letters “SW”.

One oak wood vessel was selected to give an example. Fig. 5 illustrates the dendrochronological dates which range between 1876 and 1880 – so only four rings lying between them. A photograph of a part of the vessel is shown in Fig. 6. The sapwood formation is clearly visible and marked with the letters “SW”. Table 4 gives again the dendrochronological dates and furthermore information about the number of visible sapwood rings the mean ring width and the calculated relative wood loss to the youngest stave. Taking the number of rings of sapwood usually used in Germany (approximately 20 rings, depending on the region), it is obvious that only a small amount of wood was removed in the construction process. Due to the usually used number of oak sapwood rings in Germany, which are 20 rings, it is obvious, that just very little parts of the wood can be removed due to wood-working. In the example 21 sapwood rings were counted as a maximum. The wood loss of every dated stave was calculated using the mean ring width multiplied by the number of rings missing to the youngest stave.

Although it cannot be verified how much wood was lost absolutely, this example makes clear, that hardly any wood was worked away. This statement was assumed to be generally the case for staves having about the same dendrochronological date, also if the species does not form any coloured heartwood.

### Orientation of the boards

**Fig. 7:**
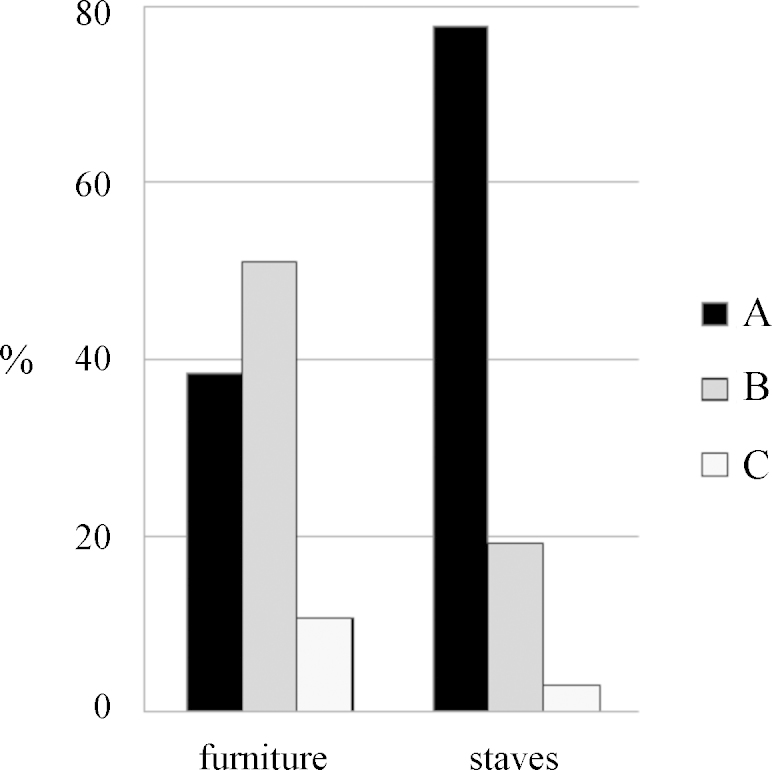
Comparison of the orientation of the boards between furniture and vessels.

Evaluating the orientation of the board within the tree, it turned out that almost 40% of the furniture was made of boards type A (radially orientated) and more than 50% of type B (between radial and tangential orientated) (see Fig. 3, Fig. 7). In vessels, however, 80% were made of type A (radially orientated).

**Fig. 8:**
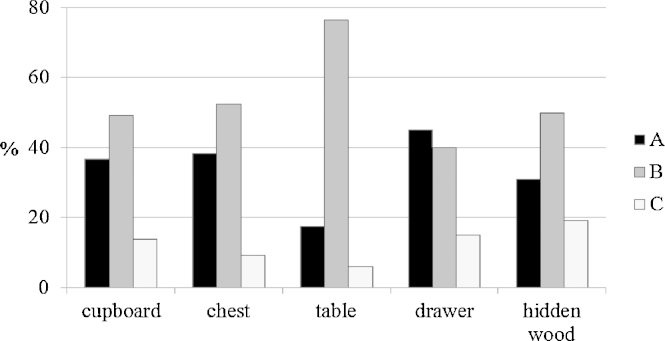
Comparing the type of boards used for different kinds of furniture.

Comparing different types of furniture, all types of boards except front boards of commodes were dominantly made from type B. Most boards of type C (tangential orientated) were found on hidden boards of cupboards or commodes. Tables were hardly ever made of type A or C (Fig. 8).

**Fig. 9:**
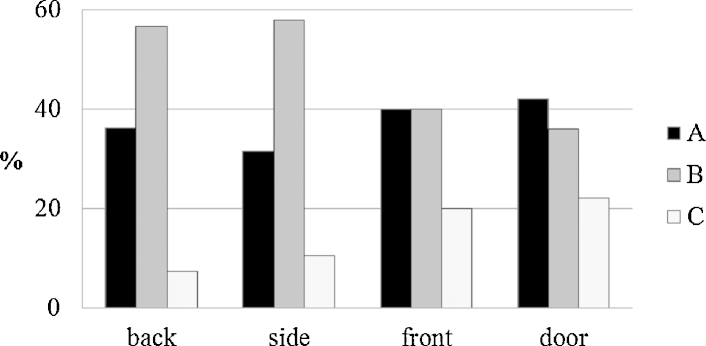
Orientation of different boards of a cupboard.

**Fig. 10:**
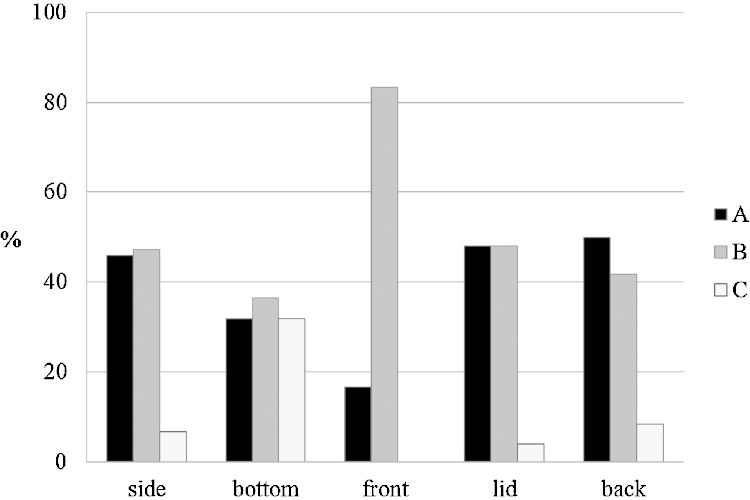
Orientation of different boards of a chest.

In Fig. 9, Fig. 10, the orientation of the boards originating from cupboards and chests are shown in detail. Interestingly back boards showed hardly any board of type C compared to the other categories and door frames showed the highest amount of board type A. For chests the front boards did not have any boards of type C and bottom boards showed almost an equal amount of boards of type A, B and C.

### Same tree origin

The calculated *t*-values for samples from the same tree are listed in Table 3. The criteria for selection was that at least one *t*-value had to meet a minimum of: *t*-values of 10 in case of Norway spruce, *t*-values of 15 in case of European larch, *t*-values of 14 in case of European oak, *t*-values of 9 in case of Stone Pine, *t*-values of 13 in case of Scots pine and *t*-values of 8 in case of Silver fir.

Among all sampled furniture and vessels, we found 10 pieces of furniture (out of 208 = 5%) and 17 vessels (out of 168 = 10%), where at least some boards or staves were cut or split out of the same tree trunk. Prefabricated staves were often stored in high stacks (Kindler, 1949), which might be the reason why not all staves within one vessel came from the same trunk. In one farmhouse we found two different vessels with staves split out of one trunk. This might be because of mixing staves after storage.

## Discussion

### Furniture

The oldest sampled furniture is dated in 1524. Furniture being more than 400 years old seems quite unusual to people living in the fast-moving 21st century. But in former times the production of furniture was led by means of durability and practicability (Moser, 1947). Wood and time for the production were valuable goods and not to be wasted. Artfully painted furniture shows the high value to the owner beyond pure utility.

Statements about seasoning times found in literature are rather diverse. Ille (1975) mentions 50 years storage time for the wood of violins. Beuting (2011), however, examined tree rings of famous violins and found a maximum period of three years. We expected seasoning time for furniture boards to be less than one generation. Indeed we found the longest time span between the dendrochronological date and the painted year to be 31 years, on average about 14 years. This time includes not only storage but also tree rings lost by wood-working. Therefore storage time was even less than one generation, often less than 10 years, in some cases only two or three years. This makes the dendrochronological dates useful for dating furniture, even if no waney edge is given. As back boards are usually rather broad and often still show a trapezoid shape, they seem to be very little shaped, whereas door frames or front panels cut with a bevel have much more wood loss through cutting and shaping. This was verified by using dendrochronology. Those boards having the most recent dendrochronological date were usually backboards. Panels having more rings missing are generally door frames or front boards. This fact makes back boards most suitable for dendrochronological dating.

Focusing on the orientation of the furniture boards, about 50% of the panels were made of type B. Tables and drawers seem to be outstanding-tables, because they have the lowest proportion of type A and C boards and drawers, because they have the highest percentage of type A boards. Focusing on cupboards, front panels and door panels have a much higher proportion of type A boards than back boards and side boards, but also a higher numbers of type C boards. Concentrating on chests, front panels did not contain any boards of type C and lids show the highest proportion of type A and the lowest proportion of type C boards.

Boards having a high width usually are not made of type C boards, because they would have rather high wood loss. Panels where dimensional stability and a flat surface was necessary for tables and chest lids, show a low proportion of type C boards, because type C boards have the highest movement due to moisture variation (Bosshard, 1975). Generally, furniture was most likely made of type A or B boards, which leads to little wood loss, too.

Boards were sawn mechanically or manually out of tree trunks of the appropriate diameter. The outermost wood represents the highest quality wood in sense of small tree rings, high density for softwood and low spiral grain (Wimmer, 1994). By cutting out a strictly radial oriented board, the smallest possible loss of the valuable outermost wood with radial orientation is given. Fig. 2 illustrates the amount of wood loss in the mentioned board types coloured in light grey. This indicates that for high quality products only little wood was wanted to be removed; therefore boards of type A had to be chosen.

### Coopered vessels

Centuries old vessels are rather astonishing – the oldest cask was dated to 1612, the oldest vat was dated to 1656. Probably they have been used for dry goods, but still they prove the high durability of wood.

Coopers produced vessels with standardised dimensions on a large scale from very early on (Radkau and Schäfer, 1987). Therefore we expect that vessels were likely than furniture to have been made by professional craftsmen, and sold on the local market, or by travelling salesmen than furniture. Unfortunately we could not find any hints in literature. Nevertheless, the standardised look of vessels or tubes and their low weight make this assumption reasonable.

If furniture and vessels are compared, the production process must have been quite different. The high percentage of type A boards proves, that vessels were split and not sawn in many cases. The close dates of the individual staves of a coopered vessel and the fact that staves had to be free of knots prove that they were split out of the outermost part of a single tree and hardly any wood was worked away. 10% of all vessels include staves split out of the same tree, compared to 5% of the samples furniture which were made of boards sawn out of the same trunk.

## Conclusion

In historical times, wood was very valuable. Furniture and vessels were in use for a long time. Wood loss through wood-working was kept to a minimum making use of the widest boards and the sections with the highest quality of the outermost wood. Storage time of boards used for furniture was mainly less than 10 years, often only two to four years. Therefore, dendrochronology is a very useful instrument to verify the age of furniture: if furniture shows a painted year, it is also possible to confirm how long after production it was painted. Staves were mostly split and not sawn out of the trunk. The small loss of wood demonstrated here makes dating without waney edge a reasonable technique for both furniture and coopered vessels.

## Figures and Tables

**Table 1:** List of the amount of identified wood species used for furniture and vessels.

	Vessels (%)	Furniture (%)
Norway spruce (*Picea abies*)	34	52
European larch (*Larix decidua*)	26	17
European oak (*Quercus* spp.)	25	
Stone pine (*Pinus cembra*)	7	14
Scots pine (*Pinus sylvestris*)	4	10
Silver fir (*Abies alba*)	4	7

**Table 2:** Number of years passed between the dendrochronological date and the painting. The objects in the first section had been painted a short period after the tree was felled; the objects under the line had been painted at a later timer or being over painted.

Object	Dendro. date	Painting	Years	mm
Cupboard	1581	1583	2	3.2
Cupboard	1773	1781	8	3.1
Cupboard	1758	1786	28	37.9
Chest	1758	1789	31	76.0
Cupboard	1798	1809	11	10.3
Cupboard	1814	1819	5	6.6
Cupboard	1811	1822	11	14.6
Table	1811	1826	15	16.5
Cupboard	1811	1832	21	10.7
Cupboard	1832	1841	9	12.4
Wine squeezer	1844	1855	11	9.1
Chest	1842	1857	15	34.8
Chest	1844	1858	14	18.3

Cupboard	1549	1847	298	
Cupboard	1598	1832	234	
Chest	1710	1883	173	
Cupboard	1729	1857	128	
Cupboard	1750	1828	78	
Cupboard	1804	1915	111	

**Table 3:** Median *t*-values after Baillie–Pilcher (*t*-value BP) and Hollstein (*t*-value H) were calculated by cross dating samples from the same tree, compared between all relevant wood species.

	*t*-Value BP	*t*-Value H
Norway spruce, alpine	10.9	11.1
Norway spruce, food hills	10.7	10.1
European oak, food hills	15.4	15.1
European larch, alpine	14.8	13.7
Stone pine, alpine	9.25	9.05
Scots pine, foodhills	12.9	16.9
Silver fir, foodhills	8.9	7.7

**Table 4:** Dated vessel which is shown in Fig. 5, Fig. 6. The wood loss refers to the stave having the youngest dendrochronological date.

Museum	Location	Stave	Wood species	Date (last ring)	Sapwood rings (*n*)	Mean ring width (mm)	Wood loss (mm)
Stübing	Burgenland	01a	Oak	**1878**	9	0.95	1.90
Stübing	Burgenland	02a	Oak	**1876**	15	0.74	2.96
Stübing	Burgenland	03a	Oak	**1880**	21	0.96	
Stübing	Burgenland	04a	Oak	Not dated	11	0.80	
Stübing	Burgenland	05a	Oak	**1879**	15	0.83	1.11
Stübing	Burgenland	06a	Oak	**1879**	16	0.91	1.15

## References

[bib0005] Bader U. (1998). Tiroler Kulturgüter – Von Truhen und Kästen.

[bib0010] Beuting M., Fraiture P. (2011). Dendro-organology? The dendrochronological method applied to music instruments. Tree Rings, Art, Archaeology. Proceedings of An International Conference.

[bib0015] Bosshard K.H. (1975). Holzkunde Band 3 – Aspekte der Holzbearbeitung und Holzverwendung.

[bib0020] Colleselli F. (1968). Tiroler Bauernmöbel.

[bib0025] Eckstein D. (2007). Human time in tree rings. Dendrochronologia.

[bib0030] Eckstein D., Wazny T., Bauch J., Klein P. (1986). New evidence for the dendrochronological dating of Netherlandisch paintings. Nature.

[bib0035] Geihofer D., Grabner M., Gelhart J., Wimmer R., Fuchsberger H. (2005). New master chronologies from historical and archaeological timber in Eastern Austria. Proceedings of the EuroDendro 2005, September 29–October 1, 2005.

[bib0040] Grünn H. (1968). Fassbinder Fassboden – Handwerk und Kunst.

[bib0045] Ille R. (1975). Eigenschaften von Fichtenresonanzholz für Meistergeigen. Holztechnologie, 16. Jahrgang, Heft 2.

[bib0050] Johann E. (1968). Geschichte der Waldnutzung in Kärnten unter dem Einfluss der Berg,- Hütten- und Hammerwerke.

[bib0055] Johann E. (1994). Regelung zur Walderhaltung und –bewirtschaftung. Österreichs Wald – Vom Urwald zur Waldwirtschaft.

[bib0105] Kindler H. (1949). Berufskunde des Handwerks, Fachliche Reihe, Folge 32. Der Handwerksberuf des Böttchers und Küfers.

[bib0060] Lipp F. (1964). Oberösterreichische Bauernmöbel – Entwicklung und landschaftliche Verbreitung der volkstümlichen Möbel in Oberösterreich von den Anfängen bis zur Gegenwart. Ausstellung Schlossmuseum Linz, Juni 1964 – Herbst 1965.

[bib0065] Lipp F. (1986). Oberösterreichische Bauernmöbel.

[bib0070] Moser O. (1947). Kärntner Bauernmöbel, I Teil: Hauskundliches und Handwerksgeschichte. Carinthia I: Geschichtliche und volkskundliche Beiträge zur Heimatkunde Kärntens, Mitteilung des Geschichtsvereins für Kärnten, 135. und 134. Jahrgang.

[bib0075] Moser O. (1949). Kärntner Bauernmöbel – Handwerksgeschichte und Frühformen von Truhe und Schrank.

[bib0080] Radkau J., Schäfer I. (1987). Holz – Ein Naturstoff in der Technikgeschichte.

[bib0085] Thun T., Alsvik E. (2009). Dendrochronological dating of four chests: a surprising result. Dendrochronologia.

[bib0110] Voigt O. (1930). Der Großböttcher und Faßberechner.

[bib0090] Waldner F., Loetscher T. (2005). Exkurs 2: Dendrochronnologische Analysen an Möbeln. Zürcher und Nordostschweizer Möbel – vom Barock bis zum Klassizismus.

[bib0095] Waldner F. (2006). Dendrochronologie der Fassdauben. Das Bürgerasyl in Stein am Rhein – Geschichte eines mittelalterlichen Spitals Schaffhauser Archäologie 7.

[bib0100] Wimmer R., Spiecker H., Kahle H.P. (1994). Structural, chemical and mechanical trends within coniferous trees. In Modelling of tree-ring development – cell structure and environment. Workshop Proceedings.

